# Three novel species of *Asterina* (*Asterinales*, *Dothideomycetes*) from Hainan Province, China

**DOI:** 10.3897/mycokeys.134.196557

**Published:** 2026-06-15

**Authors:** Yuzhe Feng, Chada Norphanphoun, Xiang-Yu Zeng

**Affiliations:** 1 Department of Plant Pathology, College of Agriculture, Guizhou University, Guiyang 550025, China College of Agriculture, Guizhou University Guiyang China; 2 Guizhou Key Laboratory of Agricultural Microbiology, Guiyang 550025, China Institute of Edible Mushrooms, Guizhou University Guiyang China; 3 Institute of Edible Mushrooms, Guizhou University, Guiyang 550025, China Guizhou Key Laboratory of Agricultural Microbiology Guiyang China

**Keywords:** *
Ascomycetes
*, black mildews, epifoliar fungi, phylogeny, taxonomy

## Abstract

*Asterina* comprises obligate biotrophic fungal pathogens that form black colonies on the surface of living leaves and derive nutrients by penetrating host epidermal cells, often resulting in tissue damage. In this study, three new species of *Asterina* from Hainan Province, China, are described based on morphological characteristics and multi-gene phylogenetic analyses (nrITS, nrLSU, and nrSSU), with phylogenetic relationships inferred by maximum likelihood (ML) and Bayesian inference (BI) to resolve their taxonomic placement. The new species *Asterina breyniicola*, *A. heyneicola*, and *A. olaciphila* were collected from living leaves of *Breynia fruticosa*, *Heynea trijuga*, and *Olax scandens*. Detailed morphological descriptions, illustrations, and a phylogenetic tree illustrating their positions are provided. These findings expand the known diversity of *Asterina* and provide integrated morphological and molecular evidence to improve species delimitation and classification within *Asterinaceae*.

## Introduction

*Asterina* represents the type genus of the family *Asterinaceae* (*Asterinales*, *Dothideomycetes*). The genus was established by Léveillé (1845), with *A. melastomatis* (Theissen 1912) designated as the type species. It is the largest genus within *Asterinaceae*, comprising over 700 species (Hofmann 2010), and is mainly distributed in tropical and subtropical regions worldwide. Initially, Saccardo (1882) placed *Asterina* in *Perisporiaceae (Perisporiales)*. However, the genus was broadly circumscribed due to morphological heterogeneity, including species with dimidiate and globose thyriothecia, and has long required taxonomic revision. The genus was first comprehensively monographed using type material of 343 described species, of which 261 were excluded as synonyms or reassigned to other genera (Theissen 1913). The remaining 82 species, together with additional newly described taxa, resulted in a revised circumscription of 108 species (Theissen 1913). Subsequently, Doidge (1919) provided detailed descriptions of spore germination, mycelium, thyriothecium, and conidial characteristics, placed *Asterina* in *Microthyriaceae*, and noted that previous uncertainties in host plant identification had led to confusion in species delimitation. *Asterinaceae* was established, and *Asterina* was transferred into this family, placing it in *Hemisphaeriales* (Hansford 1946).

Species of *Asterina* are obligately biotrophic epifoliar fungi occurring predominantly on the surface of living leaves and only rarely on living stems or other plant organs (Barr and Huhndorf 2001; Kirk et al. 2001; Taylor et al. 2005; Hofmann et al. 2010; Hyde et al. 2013). The term “obligate biotrophs” refers to a high dependency on host plants while causing minimal damage, requiring living plant cells for growth, and generally being unculturable on artificial media (Latijnhouwers et al. 2003; De Silva et al. 2016). These epifoliar fungi are comparatively understudied symbionts inhabiting the surfaces of living plants (Marasinghe et al. 2022b). They are commonly referred to as “black mildews,” forming web-like black colonies on leaf surfaces, and may cause economic damage to their hosts (Hongsanan et al. 2016; Marasinghe et al. 2022b; Marasinghe et al. 2023). Hosagoudar et al. (1997) reported that infection by *A. congesta* on sandalwood increased the production of cyclic compounds such as proline, indicating elevated stress levels in the host. They exhibit diverse infection strategies, including the formation of haustoria, hypostromata, inter- or intracellular hyphae, and appressoria. They can parasitize plants from more than 120 plant families, including herbs, trees, weeds, and economically important plants (Hofmann 2010; Hongsanan et al. 2014; Vishnu et al. 2017). Appressoria represent key structures for host penetration and nutrient acquisition in *Asterina* (Hofmann 2010; Chethana et al. 2021). Members of *Asterina* are characterized by dark brown, circular thyriothecia with stellate dehiscence; branched, dark brown, superficial mycelium with lateral appressoria; globose to subglobose, 8-spored, bitunicate asci; and dark brown ascospores with one septum and strongly constricted at the septum (Hongsanan et al. 2014). The asexual morph is referred to as *Asterostomella*, which is characterized by superficial, dark brown to brown, orbicular, pyriform pycnothyria and appressoriate superficial hyphae with brown, ovoid, aseptate conidia (Marasinghe et al. 2023). Most species in this genus are considered host-specific, at least at the host-family level (Hofmann and Piepenbring 2008), but this has not been confirmed with molecular data. In addition, they cannot be cultured on artificial media, and molecular data for these species are still scarce.

Species of *Asterina* have traditionally been described mainly based on their host association (Song et al. 2003a, 2003b; Song et al. 2004; Hofmann and Piepenbring 2008; Hosagoudar 2012). Morphological similarity among species remains high, and it is still unclear whether taxa are host species-, genus-, or family-specific. These patterns require confirmation using molecular data. At present, phylogenetic studies of *Asterina* remain limited. Hofmann et al. (2010) used sequence data from five species to provide the first phylogenetic analysis of *Asterinaceae*, supporting the family as a distinct lineage within *Dothideomycetes* and leading to its placement in *Asterinales* (Hofmann et al. 2010; Hyde et al. 2013; Boonmee et al. 2014; Wijayawardene et al. 2014). According to Index Fungorum (2026), there are 1130 taxa listed under *Asterina*, yet molecular data are available for only 16 species in NCBI. The high species number largely reflects historical reliance on host specificity for species delimitation. This assumption has not been rigorously tested using molecular data or inoculation experiments. Substantial work is therefore required to resolve species boundaries and phylogenetic relationships within the genus.

In this study, three new species isolated from living leaves of *Breynia fruticosa*, *Heynea trijuga*, and *Olax scandens* in Hainan Province, China, are introduced. Detailed descriptions of the morphological features of these species, along with their molecular characterization, are provided. These findings expand the host ranges, enrich the molecular data of *Asterina*, and increase the understanding of their diversity.

## Materials and methods

### Sample collection and morphological observation

Infected leaf specimens with black mildews were collected from Hainan Province, China, brought to the laboratory in paper envelopes, and stored in a dry environment. Specimens were examined using a stereomicroscope (Keyence VHX-7000 digital microscope, Japan) to photograph macroscopic features, including colony structure, color, and distribution of ascomata. Microscopic observations and photomicrographs were taken from materials mounted in 60% lactic acid using a compound light microscope (Zeiss Axioscope 5, Germany) equipped with an AxioCam 208 color camera and interference contrast optics. Images were captured to document the morphology, color, size, and other characteristics of microstructures such as hyphae, appressoria, thyriothecia, and spores. Measurements were taken using ZEN2 (blue edition) software, and all images used in figures were processed with Adobe Photoshop (version 2022). The specimens were deposited in the Herbarium of **IFRD** (International Fungal Research & Development Centre; Institute of Highland Forest Science, Chinese Academy of Forestry, Kunming, China) and the Herbarium of the Department of Plant Pathology, Agricultural College, Guizhou University (**HGUP**). Index Fungorum numbers were obtained by submitting the species information via the official online registration platform (Index Fungorum 2026).

### DNA extraction, PCR amplification, and sequencing

Genomic DNA of fungi was directly extracted from ascomata following the protocol described by Zeng et al. (2018). The 5.8S ribosomal RNA, along with the internal transcribed spacer (ITS), was amplified with the primer pair ITS5 and ITS4 (White et al. 1990). The partial large subunit ribosomal RNA (LSU) was amplified with the primer pair LR0R and LR5 (Vilgalys and Hester 1990; Rehner and Samuels 1994). The partial small subunit ribosomal RNA (SSU) rDNA was amplified with the primer pair NS1 and NS4 (White et al. 1990). Genomic DNA of the host plant was extracted using a Plant gDNA maxi kit (Biomiga, San Diego, California, USA) in accordance with the manufacturer’s instructions. The partial *rbcL* gene was amplified using the primer pair rbcLr and rbcLf, which were developed by Kress et al. (2009). The 5.8S rDNA, along with the ITS, was amplified with the primer pair ITS1 and ITS4 (White et al. 1990). Polymerase chain reactions (PCRs) were performed in a 20 µL reaction mixture containing 17 µL of GoldenStar T6 Super PCR Mix (1.1×), 1 µL of DNA template, and 1 µL of forward and reverse primers (10 µM/µL). Amplifications were programmed for an initial denaturation step at 95 °C, followed by 35 cycles of 30 s at 95 °C, 30 s at 54 °C (ITS), 50 s at 50 °C (LSU), 51 °C (SSU), and 50 °C (*rbcL*), and 90 s at 72 °C, with a final elongation step of 10 min at 72 °C. PCR amplification products were assayed via electrophoresis in 1% agarose. The PCR products were sent to Tsingke Biotechnology Co., Ltd., Beijing, China. The list of primers and PCR conditions for each primer pair is provided in Table 1.

**Table 1.** PCR conditions and the primers used in this study.

Locus	Primers	Sequence (5’–3’)	PCR cycles	References
ITS	ITS1	TCCGTAGGTGAACCTGCGG	(95 °C: 30 s, 54 °C:30 s, 72 °C:60 s) × 35 cycles	White et al. 1990
	ITS4	TCCTCCGCTTATTGATATGC		
	ITS5	GGAAGTAAAAGTCGTAACAAGG		
LSU	LR0R	ACCCGCTGAACTTAAGC	(94 °C: 30 s, 51 °C: 50 s, 72 °C:60 s) × 35 cycles	Rehner and Samuels 1994
	LR5	TCCTGAGGGAAACTTCG		Vilgalys and Hester 1990
SSU	NS1	GTAGTCATATGCTTGTCTC	(95 °C: 30 s, 51 °C: 50 s, 72 °C: 60 s) × 35 cycles	White et al. 1990
	NS4	CTTCCGTCAATTCCTTTAAG		
*rbcL*-a	rbcLf	ATGTCACCACAAACAGAGACTAAAGC	(95 °C: 30 s, 50 °C: 50 s, 72 °C: 60 s) × 35 cycles	Kress et al. 2009
	rbcLr	GTAAAATCAAGTCCACCRCG		

### Phylogenetic analyses

The forward and reverse sequence results of each gene were assembled using BioEdit v.7.2.5 (Hall 1999) to obtain consensus sequences. BLASTn searches were used to identify the closest matches in GenBank. The accession numbers of taxa used in the analyses are shown in Table 2. Phylogenetic analysis was conducted based on datasets including reference DNA sequences and newly generated DNA sequences using the One-click Fungal Phylogenetic Tool (OFPT), a streamlined pipeline that automates fungal phylogeny reconstruction by integrating MAFFT, TrimAl, IQ-TREE, and MrBayes (Zeng et al. 2023). The protocol was as follows: datasets of each gene region were first independently aligned with the “FFT-NS-i” strategy (based on data size) by MAFFT (Katoh and Standley 2013) and trimmed manually. The best-fit nucleotide substitution models for each dataset were then selected based on the Bayesian information criterion (BIC) from 22 common DNA substitution models with rate heterogeneity by ModelFinder (Kalyaanamoorthy et al. 2017). Afterwards, all datasets were concatenated with partition information for the subsequent phylogenetic analyses. ML with 1000 replicates was performed using ultrafast bootstrap approximation (Hoang et al. 2018) with the SH-like approximate likelihood ratio test (SH-aLRT) (Guindon et al. 2010) by IQ-TREE (Nguyen et al. 2015). The consensus tree was summarized based on the extended majority rule. BI was performed with two parallel Metropolis-coupled (one “cold” chain and three heated chains) Markov chain Monte Carlo runs by MrBayes (Ronquist et al. 2012).

**Table 2.** GenBank accession numbers of DNA sequences used in this study.

Species	Specimen voucher	GenBank accession number			References
		LSU	ITS	SSU	
*Asterina cestricola*	TH 591	GU586215	–	GU586209	Hofmann et al. (2010)
***Asterina cynometrae***	MFLU 13-0373	NG_057120	–	–	Hongsanan et al. (2016)
*Asterina fuchsiae*	TH 590	GU586216	–	GU586210	Hofmann et al. (2010)
*Asterina magnoliae*	MFLU 16-0071	MN629745	–	–	Hyde et al. (2018)
***Asterina magnoliae***	MFLU 16-0072	MG844186	–	–	Hyde et al. (2018)
*Asterina neomangiferae*	MFLU 21-0038	MZ225596	MZ225576	–	Marasinghe et al. (2022a)
*Asterina phenacis*	TH 589	GU586217	–	GU586211	Hofmann et al. (2010)
*Asterina siphocampyli*	ppMP 1324	HQ701140	–	–	Hofmann and Piepenbring (2011)
*Asterina weinmanniae*	TH 592	GU586218	–	GU586212	Hofmann et al. (2010)
*Asterina zanthoxyli*	TH 561	GU586219	–	GU586213	Hofmann et al. (2010)
*Asterostomella grewiae*	MFLU 13-0629	MN364645	–	MN364416	Hyde et al. (2020)
*Asterotexis cucurbitacearum*	PMA M-0141224	HQ610510	–	–	Guatimosim et al. (2015)
***Asterotexis cucurbitacearum***	VIC 42814	NG_057054	–	–	Guatimosim et al. (2015)
***Cirsosia mangiferae***	MFLU 21-0039	NG_148995	NR_182545	–	Marasinghe et al. (2022a)
*Cylindrohyalospora fici*	MFLUCC 19-0076	MW063243	–	–	Tennakoon et al. (2021)
*Cylindrohyalospora fici*	NCYUCC 19-0330	MW063244	–	–	Tennakoon et al. (2021)
*Inocyclus angularis*	VIC 39748	KP143732	KP273234	–	Guatimosim et al. (2015)
*Inocyclus angularis*	VIC 39749	KP143733	KP273235	–	Guatimosim et al. (2015)
***Inocyclus angularis***	VIC 39747	KP143731	KP273233	–	Guatimosim et al. (2015)
*Lembosia albersii*	MFLU 13-0377	KM386982	–	–	Hongsanan et al. (2014)
***Lembosia mimusopis***	MFLU19-1225	MN563123	–	–	Marasinghe et al. (2021)
*Lembosia mimusopis*	MFLU 19-0724	MN565982	–	–	Marasinghe et al. (2021)
***Lembosia xyliae***	MFLU 14-0004	NG_059589	–	–	Ariyawansa et al. (2015)
***Morenoina calamicola***	MFLUCC 14-1162	NG_059779	NR_154210	NG_065667	Tibpromma et al. (2017)
*Morenoina palmicola*	MFLUCC 15-0284	MK120272	–	MK120299	Hyde et al. (2019)
***Morenoina rattanica***	MFLU 24-0157	NG_245574	NR_200694	–	Zhang et al. (2024)
***Asterina heyneicola***	IFRD99055	PZ282960	PZ282969	PZ282978	This study
*Asterina heyneicola*	IFRD900-25	PZ282961	PZ282970	PZ282979	This study
*Asterina heyneicola*	HGUP 26-0002	PZ282962	PZ282971	PZ282980	This study
***Asterina breyniicola***	IFRD99056	PZ282963	PZ282972	PZ282981	This study
*Asterina breyniicola*	IFRD900-26	PZ282964	PZ282973	PZ282982	This study
*Asterina breyniicola*	HGUP 26-0003	PZ282965	PZ282974	PZ282983	This study
***Asterina olaciphila***	IFRD99057	PZ282966	PZ282975	PZ282984	This study
*Asterina olaciphila*	IFRD900-27	PZ282967	PZ282976	PZ282985	This study
*Asterina olaciphila*	HGUP 26-0004	PZ282968	PZ282977	PZ282986	This study

**Notes**: Sequences derived from holotype and epitype specimens are shown in bold.

## Results

### Molecular phylogeny

**Figure 1.**
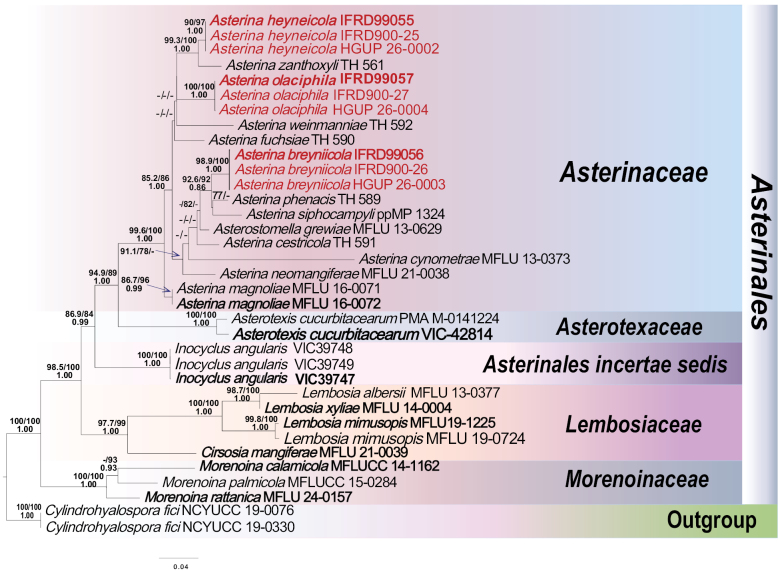
Best-scoring ML tree based on combined LSU, SSU, and ITS sequence data rooted by *Cylindrohyalospora fici* (NCYUCC 19-0076, NCYUCC 19-0030). The SH-aLRT values greater than 80%, BS values greater than 70%, and PP values greater than 0.9 are shown above the branches. Sequences obtained from holotypes and epitypes are bold. New sequences obtained in this study are in red.

Phylogenetic analysis for *Asterinales* was performed using ITS, LSU, and SSU loci. The concatenated dataset consisted of 65 strains with *Cylindrohyalospora fici* as the outgroup. The final alignment comprised 2,287 concatenated characters spanning positions 1–512 (ITS), 513–1,316 (LSU), and 1,317–2,287 (SSU). The combined dataset yielded a best-scoring tree with a final ML optimization likelihood value of −9340.022123. The phylogenetic information used for ML and BI, including model selection and sequence characteristics, is detailed in Table 3. The topology of the tree from the BI analysis was similar to that obtained from the ML analysis. Given the similarity between the ML and BI topologies, only the ML tree is shown (Fig. 1).

**Table 3.** Summary of phylogenetic parameters used in ML and BI analyses, including the best-fit substitution models, number of nucleotide positions, conserved and variable sites, and other relevant characteristics for each gene region.

Information	Gene		
	ITS	LSU	SSU
**Best fit substitution mode**	TN+F+G4	TN+F+R3	K2P+I
**Number of sequences**	16	35	17
**Number of characters**	512	804	971
**Number of constant sites**	261 (representing 50.98% of all sites	507 (representing 63.06% of all sites)	849 (representing 87.44% of all sites)
**Number of parsimony informative sites**	195	232	70
**Number of distinct site patterns**	239	339	132
**Estimated base frequencies**	A = 0.1873	A = 0.2272	equal frequencies
	C = 0.3583	C = 0.2688	
	G = 0.2975	G = 0.3311	
	T = 0.1568	T = 0.1729	
**Estimated substitution rates**	A-C = 1.0000	A-C = 1.0000	A-C = 1.0000
	A-G = 2.1715	A-G = 3.0675	A-G = 4.2096
	A-T = 1.0000	A-T = 1.0000	A-T = 1.0000
	C-G = 1.0000	C-G = 1.0000	C-G = 1.0000
	C-T = 3.7672	C-T = 8.7627	C-T = 4.2096
	G-T = 1.0000	G-T = 1.0000	G-T = 1.0000

### Taxonomy

#### Asterina breyniicola

Taxon classificationFungiValvatidaAsterinidae

Y.Z. Feng & X.Y. Zeng sp. nov.

7207984A-CB20-5539-843D-725094ACC286

Index Fungorum: IF905324

Fig. 2

##### Etymology.

In reference to the host species name.

**Figure 2.**
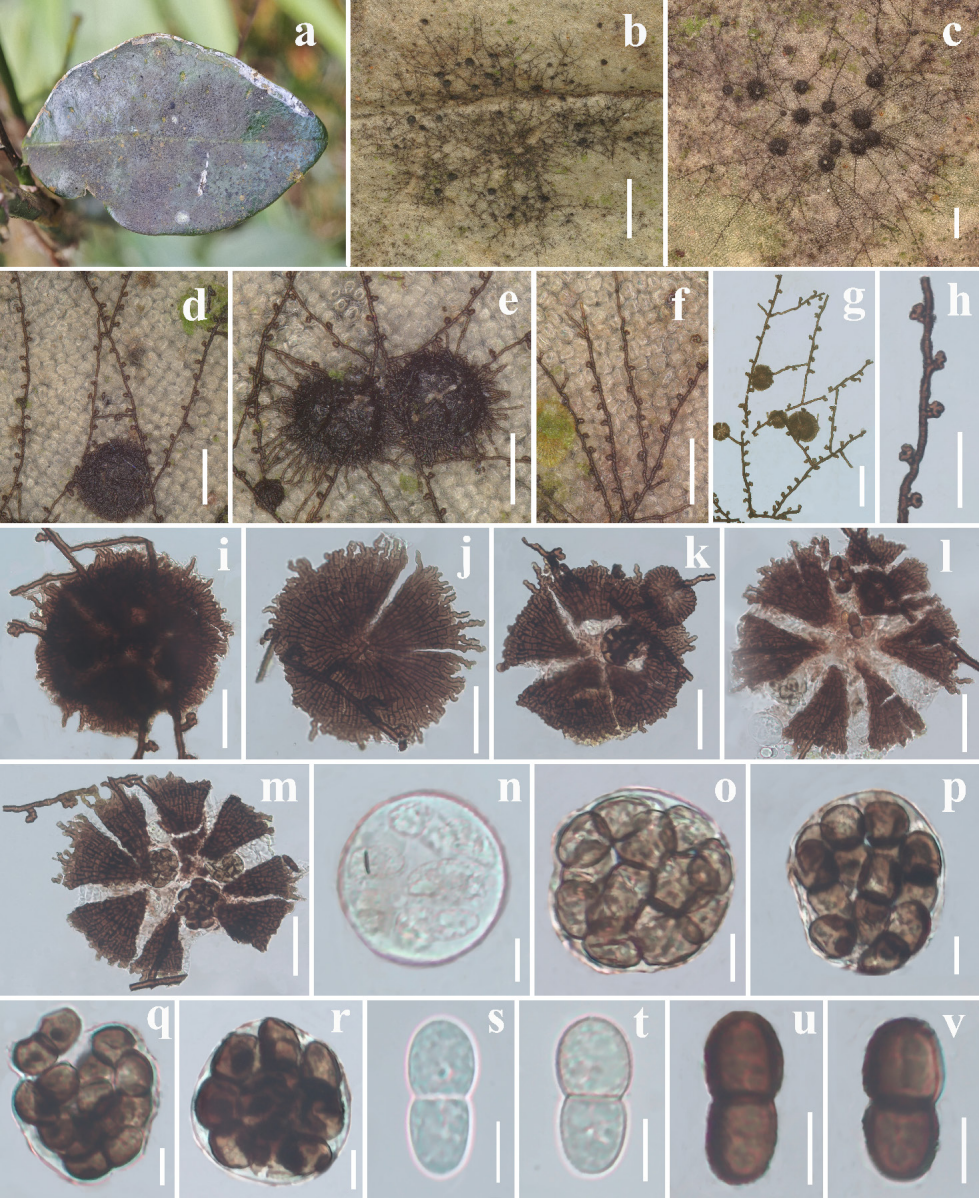
*Asterina breyniicola* (IFRD99056) **a**. Fungal colonies on fresh leaf of *Breynia fruticosa*; **b, c**. Blackened web-like colonies on leaf surface; **d, e**. Thyriothecia and appressoria; **f–h**. Appressoria; **i–m**. Squash mount of thyriothecia; **n**. Immature asci; **o–r**. Mature asci; **s–v**. Immature to mature ascospores. Scale bars: 1000 μm (**b**); 250 μm (**c**); 100 μm (**d–g**); 50 μm (**h–m**); 10 μm (**n–v**).

##### Description.

***Parasitic*** on the upper surface of living leaves, forming blackened circular to irregular and minute areas, 1–5 mm diam. ***Superficial hyphae*** abundant, brown-black, loosely reticulate, spreading over the host surface, straight to substraight, branching opposed to irregular at acute angles, smooth, septate, ***hyphal cells*** cylindrical, 22.9–34.5 × 2.4–4.6 (x̄ = 27.5 × 3.6 µm, *n* = 20). ***Appressoria*** numerous, 1-septate, digitate, multilobate with 3 or more large lobes and various smaller lobes, straight or hooked, not sessile, at base narrow, with a stalk cell (sparsely without), stalk cell short cylindrical, alternate or unilateral, 9.2–13.8 × 8.7–11.9 µm (x̄ = 11.7 × 9.9 µm, *n* = 60), brown. **Sexual state: *Thyriothecia*** 130.3–210.8 µm diam. (x̄ = 169.2 µm, *n* = 40), superficial, developing beneath the superficial mycelium, scattered to confluent on host surface, circular, flattened, dark brown, easily removed from host surface, with star-like opening, fissure at the center, sometimes opening widely to expose mature and immature asci. ***Upper wall*** comprising rows of neatly arranged, parallel, radiating cells, which may be branched at the outer rim, from which hyphae strands develop. Hamathecium lacking pseudoparaphyses. ***Asci*** 35.9–45.4 × 32.9–41.3 µm (x̄ = 39.7 × 37.2 µm, *n* = 10), octosporous, bitunicate, globose to subglobose, lacking a pedicel. ***Ascospores*** 21.7–25.8 × 10.6–12.6 µm (x̄ = 24.5 × 11.4 µm, *n* = 10), overlapping 2–3-seriate, conglobate, hyaline, 1-septate, strongly constricted at the septum, upper cell slightly larger than lower cell, hyaline when immature, dark brown at maturity, smooth-walled. **Asexual state**: Unknown.

##### Material examined.

China • Hainan Province, Ledong Li Autonomous County, Jianfengling National Forest Park; 18°39'59"N, 108°44'17"E; on living leaves of *Breynia fruticosa* (*Phyllanthaceae*); 6 November 2025; Yu-Zhe Feng; IFRD99056 (holotype) • ibid IFRD900-26 (isotype) • *ibid*; HGUP 26-0005.

##### Notes.

This collection represents the first *Asterina* species from *Breynia* for which DNA sequence data are available. There are two *Asterina* species previously reported on *Breynia*, namely *A. breyniae* (Sydow and Sydow 1917) and *A. breyniaecola* (Saccardo et al. 1926), but the original description of *A. breyniaecola* by Trotter (Saccardo et al. 1926) is not accessible. Furthermore, *A. breyniae* has smaller thyriothecia (150–250 vs. 130.3–210.8 µm) and longer asci (45–55 × 35.9–45.4 vs. 35.9–45.4 × 32.9–41.3 µm) than the present collection (Sydow and Sydow 1917). Phylogenetically, the new collection clusters with *A. phenacis* with high bootstrap support but differs by 2% (13/561 nucleotides, 2 gaps) and 2% (8/461 nucleotides, 2 gaps) in the LSU and SSU regions, respectively. ITS comparisons were omitted due to the absence of reference sequences for *A. phenacis* in public databases. Morphologically, *A. phenacis* has larger appressoria (11–16 × 8–9 vs. 9.2–13.8 × 8.7–11.9 µm), smaller thyriothecia (60–125 vs. 130.3–210.8 µm), smaller asci (35–40 × 25–30 vs. 35.9–45.4 × 32.9–41.3 µm), and smaller ascospores (15–18 × 7.5–10 vs. 21.7–25.8 × 10.6–12.6 µm) (Hofmann and Piepenbring 2008) (Table 4).

**Table 4.** Morphological comparison of the newly described *Asterina* species and related taxa.

Species	Hyphal cell (μm)	Appressoria (μm)	Thyriothecia (μm)	Asci (μm)	Ascospores (μm)	Pycnothyriospores (μm)	Reference
***Asterina heyneicola***	13.3–20.6 × 3.3–6.1	8.1–10.2 × 4.4–7.1	149.2–266.4	41.7–61.6 × 39.9–55.1	24.8–31.6 × 11.2–16.5	–	This study
*Asterina zanthoxyli*	17–27 × 4–5	10–13 × 5–6	194–235	36–51	29–33 × 14–16	–	Hofmann et al. 2010
*Asterina aglaiae*	16–23 × 4–6	8–13 × 5–15	up to 120	up to 30	20–28 × 11–13	–	Thimmaiah et al. 2013
*Asterina chukrasiae*	19–23 × 3–5	4–6 × 6–7	up to 100	up to 30	14–20 × 11–13	–	Thimmaiah et al. 2013
*Asterina cipadessae*	20–27 × 2.5–3.5	8–12 × 3–5	up to 250	28	20–24 × 9–13	–	Thimmaiah et al. 2013
*Asterina trichiliae*	16–23 × 4.5–6	8–12 × 5–7	up to 200	up to15	21–28 × 12–15	–	Thimmaiah et al. 2013
*Asterina phenacis*	17–31 × 4–5	11–16 × 8–9	60–125	35–40 × 25–30	15–18 × 7.5–10	–	Hofmann and Piepenbring 2008
***Asterina breyniicola***	22.9–34.5 × 2.4–4.6	9.2–13.8 × 8.7–11.9	130.3–210.8	35.9–45.4 × 32.9–41.3	21.7–25.8 × 10.6–12.6	–	This study
*Asterina breyniae*	5–7 (wide)	15–18 × 10–12	150–250	45–55 × 30–38	21–25 × 10–11	–	Sydow and Sydow 1917
***Asterina olaciphila***	22.1–31.1 × 6.2–8.9	10.3–15.1 × 6.5–11.0	120.2–225.2	51.9–72.8 × 49.9–73.9	33.8 × 39.8–16.9 × 21.6	20.2–29.6 × 16.7–23.8	This study
*Asterina olacicola*	19–23 × 8–10	11–13 × 6–7	up to 160	up to 40	27–36 × 14–19	19–23 × 15–17	Hosagoudar 2012
*Asterina weinmanniae*	23–35 × 5–6	10–14 × 5–8	175–227	33–43	28–33 × 14–16 (upper) / 12–14 (lower)	–	Hofmann et al. 2010
*Asterina olacis*	18–28 × 5.5–7.5	6–17 × 5–8	up to170	–	31–37 × 16–18	20–27 × 16–18	Song et al. 2003b

**Notes**: Comparison taxa were selected based on their close phylogenetic relationships and host associations with the newly described species.

#### Asterina heyneicola

Taxon classificationFungiValvatidaAsterinidae

Y.Z. Feng & X.Y. Zeng sp. nov.

8778EC93-EB36-55CB-A200-5EEC6ACFC69A

Index Fungorum: IF905323

Fig. 3

##### Etymology.

In reference to the host species name.

**Figure 3.**
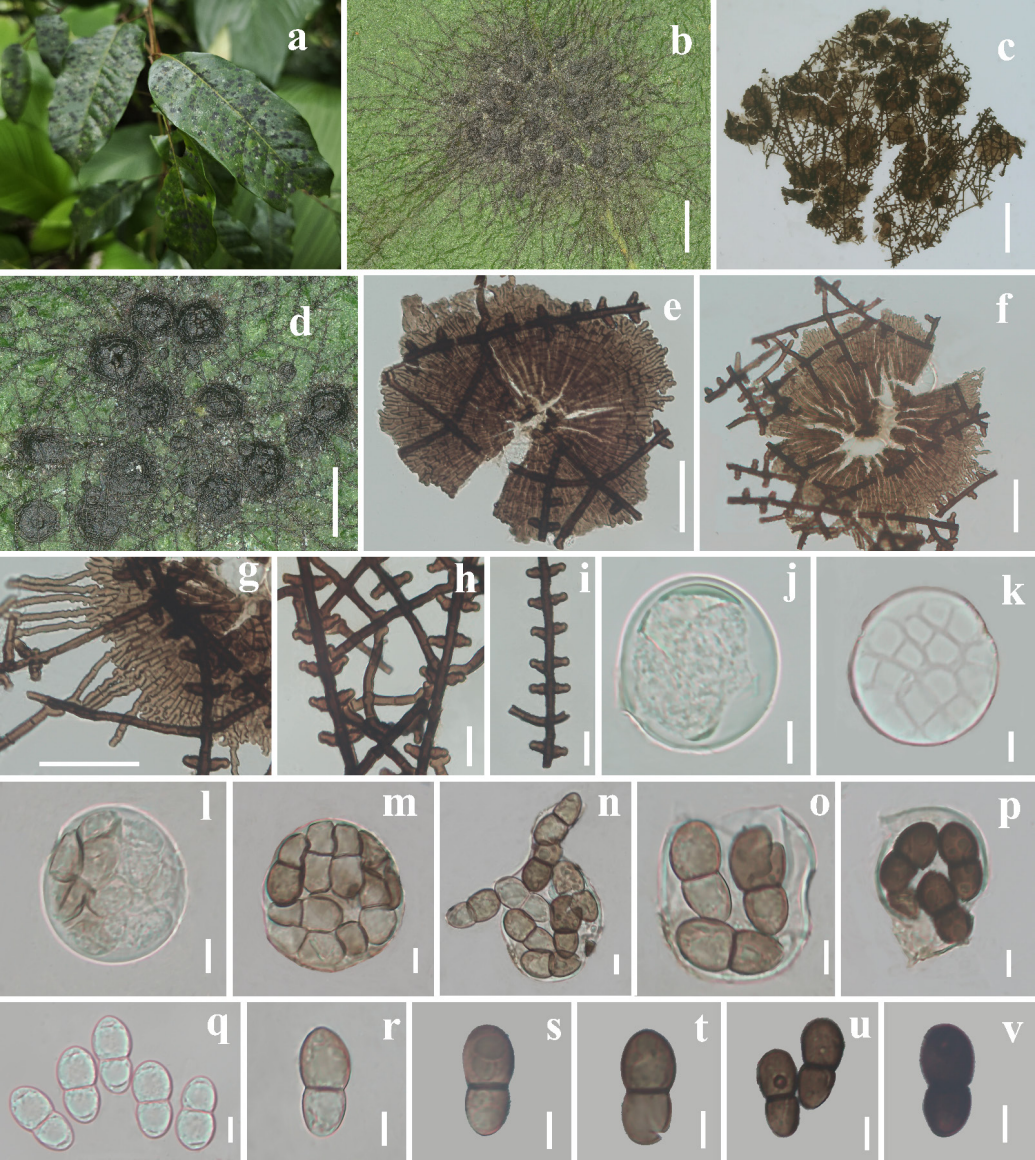
*Asterina heyneicola* (IFRD99055) **a**. Fungal colonies on fresh leaf of *Heynea trijuga*; **b, c**. Web-like colonies on leaf surfaces; **d**. Appearance of colony with thyriothecia on the surface of leaves; **e, f**. Squash mount of thyriothecia; **g**. Squash mount of thyriothecial upper wall; **h, i**. Appressoria; **j–l**. Immature asci; **m–p**. Mature asci; **q–v**. Immature to mature ascospores. Scale bars: 500 μm (**b**); 200 μm (**c, d**); 50 μm (**e–g**); 20 μm (**h, i**); 10 μm (**j–v**).

##### Description.

***Parasitic*** on living leaves. Colonies circular to irregular, epiphyllous, single to confluent, black, 1–3 mm diam. ***Superficial hyphae*** abundant, brown-black, loosely reticulate, spreading over the host surface, straight to substraight, branching opposed to irregular at acute angles to wide angles, smooth, septate, ***hyphal cells*** cylindrical, 13.3–20.6 × 3.3–6.1 (x̄ = 15.9 × 5.0 µm, *n* = 40). ***Appressoria*** lateral, mostly opposite, rarely alternate, 1-celled, gourd-shaped, subantrorse to subparallel, light brown, 8.1–10.2 × 4.4–7.1 µm (x̄ = 9.0 × 5.7 µm, *n* = 60). **Sexual state: *Thyriothecia*** 149.2–266.4 µm diam. (x̄ = 191.9 µm, *n* = 40), flattened, developing beneath the superficial mycelium, gregarious, single to confluent, light brown to dark brown, with hyphae of different lengths at the margin, borne on a thin, hyaline basal stroma, stellately dehiscing with a slightly opened center when mature, sometimes opening widely to expose mature and immature asci, and easily detached from the host surface. ***Upper wall*** linear, with compressed hyphae arranged radially from the center toward the margin, and fringed at margins. Hamathecium lacking pseudoparaphyses. ***Asci*** 41.7–61.6 × 39.9–55.1 µm (x̄ = 46.7 × 51.4 µm, *n* = 10), 8-spored, globose to subglobose, bitunicate, lacking a pedicel. ***Ascospores*** 24.8–31.6 × 11.2–16.5 µm (x̄ = 28.9 × 14.4 µm, *n* = 60), overlapping, with 2-layered wall, one pseudoseptate, strongly constricted at the septum, wall smooth, hyaline when immature, becoming brown to black when mature, upper cell subglobose, lower cell slightly longer and narrower, oval to obovoid. **Asexual state**: Unknown.

##### Material examined.

China • Hainan Province, Qiongzhong Li and Miao Autonomous County, Baihualing Ridge; 19°0'21"N, 109°49'29"E; onliving leaves of *Heynea trijuga* (*Meliaceae*); 8 November 2025; Yu-Zhe Feng leg.; IFRD99055 (holotype) • ibid IFRD900-25 (isotype) • *ibid*; HGUP 26-0003.

##### Notes.

This represents the first record of *Asterina* on *Heynea (Meliaceae)*. Morphological comparisons were conducted with *Asterina* species previously reported from other genera in *Meliaceae*, namely *A. aglaiae*, *A. chukrasiae*, *A. cipadessae*, and *A. trichiliae* (Hosagoudar 2012). The new collection resembles *A. aglaiae* and *A. trichiliae*, but *A. heyneicola* differs in having larger thyriothecia (up to 266.4 µm) and asci (up to 55.1 µm). Phylogenetically, the new collection is sister to *A. zanthoxyli*, but with 2% (13/537 nucleotides, no gaps) and 1% (6/458 nucleotides, no gaps) differences in the LSU and SSU regions, respectively. ITS sequences are unavailable for *A. zanthoxyli*, precluding their inclusion in the phylogenetic analysis. Morphologically, *A. zanthoxyli* differs in having amphigenous colonies, predominantly alternate appressoria, and longer hyphal cells (17–27 vs. 13.3–20.6 µm) (Hofmann et al. 2010) (Table 4).

#### Asterina olaciphila

Taxon classificationFungiValvatidaAsterinidae

Y.Z. Feng & X.Y. Zeng sp. nov.

B84E57B1-8890-5C37-A3AA-F7BC198A6A55

Index Fungorum: IF905325

Fig. 4

##### Etymology.

In reference to the host species name.

**Figure 4.**
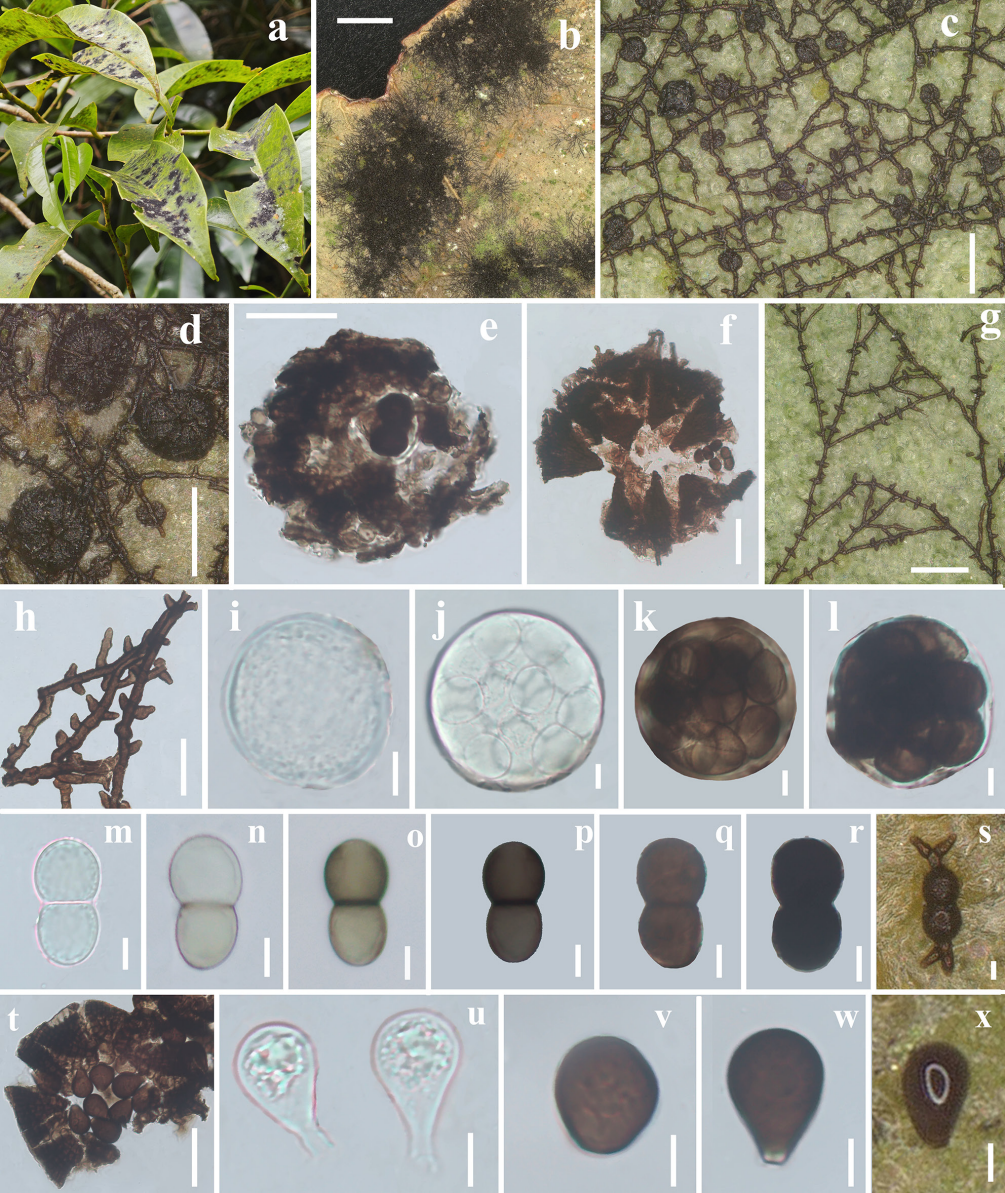
*Asterina olaciphila* (IFRD99057) **a**. Fresh leaf of *Olax scandens*; **b**. Blackened colonies on leaf surface; **c, d**. Web-like appearance of colonies on the leaf surface; **e, f**. Squash mount of thyriothecia; **g, h**. Appressoria on hyphae; **i, j**. Immature asci; **k, l**. Asci at maturity; **m–r**. Immature to mature ascospores; **s**. Germinated ascospore; **t**. Pycnothyria; **u–x**. Pycnothyriospores. Scale bars: 2000 μm (**b**); 100 μm (**c, d, g**); 50 μm (**e, f, h, t**); 10 μm (**i–s, u–x**); 10 μm (**j–v**).

##### Description.

***Parasitic*** on living leaves, forming blackened, dense, web-like areas, colonies amphigenous, circular or irregular, single to confluent, dense, 2–8 mm diam. ***Superficial hyphae*** numerous, brown-black, closely reticulate, spreading over the host surface, straight to curved, branching opposed to irregular at acute angles, smooth, septate, ***hyphal cells*** cylindrical, 22.1–31.1 × 6.2–8.9 (x̄ = 26.5 × 7.2 µm, *n* = 30). ***Appressoria*** numerous, lateral, unicellular, ampulliform or slightly conical, with the top relatively shield-like, opposite or irregular, subantrorse to subretrorse, brown, 10.3–15.1 × 6.5–11.0 µm (x̄ = 12.6 × 7.9 µm, *n* = 100). **Sexual state: *Thyriothecia*** 120.2–225.2 µm diam. (x̄ = 154.4 µm, *n* = 20), superficial, flattened or subglobose, densely gregarious, single to confluent, stellately dehisced and widely opened at the center. ***Asci*** 51.9–72.8 × 49.9–73.9 µm (x̄ = 65.2 × 64.5 µm, *n* = 20), octosporous, bitunicate, globose, lacking a pedicel. ***Ascospores*** 33.8–39.8 × 16.9–21.6 µm (x̄ = 36.9 × 19.2 µm, *n* = 60), 2–3-seriate in the ascus, ovalellipsoid, 1-septate, constricted at the septum, hyaline when immature, becoming brown to dark brown at maturity, smooth-walled. **Asexual state: *Pycnothyria*** many, similar to thyriothecia, smaller; orbicular, 75.6–154.3 µm (x̄ = 104.9 µm, *n* = 20) in diameter. ***Pycnothyriospores*** many, pyriform, brown, 20.2–29.6 × 16.7–23.8 (x̄ = 25.9 × 19.6 µm, *n* = 50) µm, wall smooth.

##### Material examined.

China • Hainan Province, Ledong Li Autonomous County, Jianfengling National Forest Park; 18°39'59"N, 108°44'17"E; on surface of leaves of *Olax scandens* (*Olacaceae*); 6 November 2025; Yu-Zhe Feng leg.; IFRD99057 (holotype) • ibid IFRD900-27 (isotype) • *ibid*; HGUP 26-0007.

##### Notes.

This collection represents the first *Asterina* species on *Olax* for which DNA sequence data are available. Two species of *Asterina* have previously been reported on *Olax* species, namely *A. olacicola* (Hosagoudar 2012) and *A. olacis* (Song et al. 2003b), but the present taxon differs from *A. olacis* in possessing larger thyriothecia (170 vs. 225.2 µm) and smooth ascospores (Song et al. 2003b). It differs further from *A. olacicola* by having opposite appressoria, larger asci (73.9 vs. 40 µm), and pycnothyriospores (20.2–29.6 × 16.7–23.8 vs. 19–23 × 15–17 µm) (Hosagoudar 2012). Phylogenetically, the present taxon is closely related to *A. weinmanniae* and *A. zanthoxyli* (Hofmann et al. 2010). It shows a 7% difference (38/564 nucleotides, 3 gaps) in the LSU region and a 2% difference (8/418 nucleotides, no gaps) in the SSU region from *A. weinmanniae*. In comparison with *A. zanthoxyli*, it differs by 5% (28/561 nucleotides, 4 gaps) in the LSU region and 2% (11/456 nucleotides, 3 gaps) in the SSU region. The ITS region was not included in the comparison because the related taxa lack ITS sequence data available for alignment. Morphologically, *A. olaciphila* is distinguished by having larger asci and ascospores and opposite appressoria, as detailed in Table 4.

## Discussion

*Asterina* represents a poorly studied genus of epifoliar fungi, with molecular data unavailable for most of its species (Marasinghe et al. 2022a). Based on an integrative taxonomic approach combining morphology, phylogeny, and host association, *A. breyniicola*, *A. heyneicola*, and *A. olaciphila* are introduced as new species. All new species form independent lineages in the phylogenetic trees and show consistent differences from closely related taxa, supporting their recognition as distinct taxa. These findings expand the known diversity of *Asterina* and provide essential molecular data for future systematic studies of *Asterinaceae*. Phylogenetic analyses based on combined ITS, LSU, and SSU sequence data placed the three new species in *Asterina* with well-supported clades. This topology is broadly consistent with recent molecular studies of *Asterinales*, although previous studies have primarily relied on single-gene (LSU) (Hongsanan et al. 2020; Marasinghe et al. 2021; Tennakoon et al. 2021; Jayawardena et al. 2022) or two-gene datasets (ITS-LSU and LSU-SSU) (Hyde et al. 2019; Marasinghe et al. 2022a; Marasinghe et al. 2023). Previous phylogenetic analyses have shown that several species currently assigned to *Asterina*, such as *A. melastomatis* and *A. chrysophylli*, do not group within the main *Asterina* clade (Marasinghe et al. 2021; Tennakoon et al. 2021; Jayawardena et al. 2022). The ordinal and familial placement of these taxa remains controversial: they have been variously treated as *Asterinales* sensu stricto (Jayawardena et al. 2022), *Asterinales* incertae sedis (*Thyrinulaceae*) (Tennakoon et al. 2021), and *Asterinaceae* sensu lato (Marasinghe et al. 2021) by different authors. Due to this taxonomic uncertainty and their considerable phylogenetic distance from the *Asterina* clade, some researchers have excluded these taxa from ingroup analyses (Hongsanan et al. 2020; Marasinghe et al. 2022a; Marasinghe et al. 2023). In the present study, several taxa (*A. mandaquiensis* VIC 42824, *A. brigadeirensis* VIC 44217, *A. lopi* VIC 44219, *A. melastomatis* VIC 42822, and *A. chrysophylli* VIC 42823) formed two distinct clades separate from the main *Asterina* clade and were therefore excluded from the ingroup. This topology may reflect DNA contamination or inhibitory effects associated with direct extraction from thyriothecia. Alternatively, these results may indicate that the current circumscription of *Asterina* is not monophyletic and requires substantial taxonomic revision. Similar phylogenetic inconsistencies suggest that traditional morphology-based classifications may obscure natural evolutionary relationships within *Asterinales*. Consequently, further molecular evidence is essential to refine the classification of this genus.

Species of *Asterina* are obligate biotrophs that cannot be cultured (Firmino and Pereira 2021). Molecular data for these species must therefore be obtained through direct DNA extraction from fresh fruiting structures. Furthermore, direct DNA extraction remains technically challenging due to their tiny fruiting structures, which are often intermixed with other fungi on leaf surfaces (Renard et al. 2020). DNA extraction from old herbarium specimens is generally unsuccessful, although it has been achieved in a few cases (O’Gorman et al. 2008; Telle and Thines 2008; Hawksworth 2013; Guatimosim et al. 2015). Therefore, most previous studies have relied solely on morphology, and many taxa remain without molecular data (Firmino et al. 2016). One major limitation in current studies of *Asterinales* is the insufficient authenticated sequence data in public databases, particularly for type or reference species. In addition, phylogenetic analyses are often based on a limited number of loci because obtaining high-quality DNA from obligate biotrophic fungi remains difficult, and ITS sequences are unavailable for many taxa. Consequently, ITS-based comparisons were not possible for some closely related taxa because corresponding ITS reference sequences are still unavailable in public databases. Future studies incorporating multilocus datasets will therefore be essential for resolving evolutionary relationships within *Asterinales*.

The traditional taxonomic framework proposed by Hansford (1946), primarily based on host specificity and morphological characteristics, continues to be widely applied in recent studies (Hofmann and Piepenbring 2008; Hongsanan et al. 2014; Bhise et al. 2023). Given the current paucity of molecular data, species delineation remains largely dependent on morphological analysis, particularly comparative studies with previously described congeners associated with identical or phylogenetically proximate host plants (Ouyang et al. 1995; Song et al. 2003c; Hosagoudar 2012). Since most species in *Asterina* are known only from their sexual morphs, species delimitation relies heavily on traits such as the arrangement, shape, and dimensions of appressoria; the size of thyriothecia; and the size of ascospores. For example, *A. horsfieldiicola* and *A. pycnanthis* can be distinguished based on differences in the arrangement of appressoria and the size of ascospores (Sydow 1938; Song et al. 2003a), while *A. flacourtiaceicola* and *A. delicata* differ notably in that the former has smaller ascospores (Doidge 1919; Song et al. 2003a). Additionally, *A. lauracearum* and *A. cinnamomi* are differentiated by the latter having globose and lobed appressoria (Sydow 1923; Ouyang et al. 1995; Song et al. 2003c). Morphological comparisons among related *Asterina* species are provided in Table 4. The results indicate that some morphological features, particularly the arrangement and shape of appressoria and ascospore dimensions, remain useful for distinguishing closely related taxa. However, morphologically similar taxa are not necessarily phylogenetically related, suggesting that certain morphological traits may exhibit convergent evolution or possess limited phylogenetic signal. Therefore, morphology alone may be insufficient for reliable species delimitation in *Asterina*, although it remains taxonomically informative when combined with molecular phylogenetic evidence.

Host association has historically played a central role in the taxonomy of *Asterina*, and many species were described primarily on the basis of host identity and morphology. Previous studies suggested that many species of *Asterina* may be host-specific, at least at the host-family level (Hofmann and Piepenbring 2008). However, most historical classifications were established without molecular evidence, and Vishnu et al. (2017) questioned whether host specificity in *Asterinales* has been adequately demonstrated. Because the three newly described species were collected from different host taxa, the present study cannot directly assess host specificity. Nevertheless, the molecular data generated here provide an important framework for future studies evaluating the taxonomic significance of host association in *Asterina*. Additional sampling across hosts and geographic regions will be necessary to determine the extent to which host association reflects species boundaries and evolutionary relationships within the genus.

Species delimitation in obligate biotrophic fungi remains particularly challenging because these fungi are unculturable and often yield only limited molecular data. In *Asterinales*, commonly used ribosomal markers such as LSU and SSU are relatively conserved, and no universal nucleotide divergence threshold has been established for species delimitation. For example, the accepted species *A. phenacis* and *A. siphocampyli* differ by only approximately 2% (13/562 nucleotides, 1 gap) in LSU sequence data, despite being recognized as distinct taxa (Hofmann et al. 2010; Hofmann and Piepenbring 2011). The absence of ITS sequences for both species and SSU data for *A. siphocampyli* further reflects the limited molecular resources currently available for many taxa in the genus. These observations indicate that species boundaries in *Asterina* cannot be evaluated solely on sequence divergence but should instead be assessed using multiple lines of evidence. In the present study, species recognition is supported by independent phylogenetic placement together with morphological differences and host associations. Therefore, an integrative taxonomic approach remains essential for robust species delimitation in *Asterina*.

## Supplementary Material

XML Treatment for Asterina breyniicola

XML Treatment for Asterina heyneicola

XML Treatment for Asterina olaciphila

## Additional information

### Artificial Intelligence (AI) use

The authors accept full responsibility for the content of the manuscript, including the disclosure of any use of AI.

No AI tools were used in the preparation of this manuscript.

### Author ORCIDs

Y. Feng https://orcid.org/0009-0000-8832-7355

C. Norphanphoun https://orcid.org/0000-0002-5756-7206

X.-Y. Zeng https://orcid.org/0000-0003-1341-1004

### Data availability

All of the data that support the findings of this study are available in the main text.
